# Preventive Effect of Blueberry Extract on Liver Injury Induced by Carbon Tetrachloride in Mice

**DOI:** 10.3390/foods8020048

**Published:** 2019-02-01

**Authors:** Bihui Liu, Yuan Fang, Ruokun Yi, Xin Zhao

**Affiliations:** 1 Chongqing Collaborative Innovation Center for Functional Food, Chongqing University of Education, Chongqing 400067, China; liubh@foods.ac.cn (B.L.); yirk@cque.edu.cn (R.Y.); 2 Chongqing Engineering Research Center of Functional Food, Chongqing University of Education, Chongqing 400067, China; 3 Chongqing Engineering Laboratory for Research and Development of Functional Food, Chongqing University of Education, Chongqing 400067, China; 4 College of Biological and Chemical Engineering, Chongqing University of Education, Chongqing 400067, China; fangyuan@foods.ac.cn

**Keywords:** blueberry, polyphenol, liver injury, carbon tetrachloride, mice

## Abstract

The blueberry is a common fruit that is rich in nutritional value and polyphenol substances. In this study, the blueberry polyphenol content in extract was analysed by spectrophotometry. The results showed that the blueberry polyphenol content in the extract reached 52.7%. A mouse model of liver injury induced by carbon tetrachloride (CCl_4_) was established to study the preventive effect of blueberry extract (BE) on liver injury in mice and the experimental animals were examined using biochemical and molecular biological methods. Aspartate aminotransferase (AST) and alanine aminotransferase (ALT) are important clinical liver function indicators; the changes of triglyceride (TG) and total cholesterol (TC) are observed after liver injury; interleukin-6 (IL-6), tumour necrosis factor-α (TNF-α) and interferon-γ (IFN-γ) are important inflammatory indexes; superoxide dismutase (SOD) activity and thiobarbituric acid reactive substances (TBARS) are important changes of oxidative stress indexes. The in vivo animal experiment results showed that BE decreased the liver index of mice with liver injury, BE could reduce the AST, ALT, TG and TC levels and also could reduce the serum cytokine IL-6, TNF-α and IFN-γ levels in mice with liver injury. Moreover, BE increased the SOD activity and decreased the TBARS level in the gastric tissues of mice with liver injury. After treatment with the highest concentration of BP in liver injury mice, these levels returned close to those obtained after treatment with the standard drug of silymarin. Detection of messenger RNA (mRNA) in liver tissue showed that BE upregulated the Cu/Zn-SOD, Mn-SOD and chloramphenicol acetyltransferase (CAT) expression levels and downregulated cyclooxygenase (COX)-2 expression. The effect of BE on mice with liver injury was positively correlated with the BE concentration and was similar to that of silymarin, which is a drug for liver injury, suggesting that BE had a good preventive effect on liver injury. Thus, BE rich in polyphenols is a bioactive substance with value for development and utilization.

## 1. Introduction

Blueberry belongs to family Ericaceae and genus *Vaccinium* L. It is a perennial deciduous or evergreen shrub. The fruit is dark blue with white frost and is nearly round, has a delicate pulp, tastes sweet and sour and is rich in nutrients. The range of blueberry processing and applications is not extensive [1]. Blueberries are rich in nutrients as well as conventional sugars, acids and vitamin C (VC) and contain plenty of vitamin E (VE), vitamin A (VA), vitamin B (VB), superoxide dismutase (SOD), arbutin, proteins, anthocyanin, dietary fibre and many mineral elements, such as K, Fe, Zn, Ca, etc [2]. The polyphenol content in the blueberry is the highest among fruits and vegetables and mainly includes anthocyanins, procyanidins, flavonoids, tannins and phenolic acids. There is clear evidence that blueberries contain catechins, lycopene, pycnogenol and resveratrol, although the presence of some phenolic flavonoids is not fully proven [3]. Some studies have noted that the main component of polyphenols in blueberry leaves is oligoanthocyanidin, although chlorogenic acid, quercetin glycoside, flavonoid glycoside, flavanol, catechin and other polyphenols are also present [4]. Blueberries have antimutagenic, anti-tumour, antiviral and antioxidative effects, which are mainly mediated by the high polyphenol content [5]. Studies have shown that the scavenging ability of blueberry polyphenol extract for hydrogen peroxide, hydroxyl radical and oxygen free radical increases with the increase in the blueberry polyphenol concentration and that the reducing capacity and scavenging ability for superoxide anion reach the level of VC [6]. Moreover, the anti-inflammatory mechanism of blueberry polyphenol can interrupt oxidative stress via the antioxidant stress pathway, which is accomplished mainly through promoting arachidonic acid metabolism, phagocytic cell accumulation at the inflammatory loci under action of proinflammatory factors and release of a large number of reactive oxygen species (ROS). Free radicals can cause lipid peroxidation and promote lysosome release, thereby reducing the release of various inflammatory mediators [7].

Free radicals are intermediate products of energy transfer, free radicals participate in various physiological and biochemical reactions in the human body and the dynamic balance of the free radical content has great significance for maintaining the stability and health of the human internal environment. Now methods such as antioxidant/oxidant balance (AOB) are used to assess the oxidative balance, which is directly related to the health of the body [8]. When stimulated by external environmental factors or increasing age, the accumulation rate of free radicals in cells is greater than the clearance rate, resulting in oxidative stress; this stress is manifested as tumourigenesis of cells, tissues and organs, loss of function, chromosomal mutations and even death [9]. Carbon tetrachloride (CCl_4_) can cause liver injury and the main mechanism is related to CCl_4_ itself and its free radical metabolites. CCl_4_ is metabolized by cytochrome P4502El in the liver to form toxic metabolites [10]. The lysozyme effect of CCl_4_ itself can lead to hepatocyte damage. The liver damage caused by the free radicals produced by CCl_4_ is thought to be the main mechanism [11]. Oxidative stress induced by ROS can damage hepatocytes to a certain extent and may play a key role in the pathogenesis of various liver injuries. ROS are mainly derived from adenosine triphosphate (ATP) production by the mitochondrial respiratory chain complex via electron transfer. The liver is rich in mitochondria and therefore, the liver is the main organ attacked by ROS. Oxidative stress can cause acute liver damage and affect the body [12].

Blueberry polyphenol had been paid attention to and the research on them had been gradually carried out. Previous studies focused on the extraction methods of blueberry, it was found that the content of anthocyanins and total polyphenols in the extract was higher than other extraction methods with the assistance of hydrodynamic cavitation [13,14]. In this study, blueberry extract (BE) was extracted, and the preventive effect of BE on acute liver injury in mice was evaluated in a CCl_4_-induced liver injury model. The sera and liver tissues of experimental mice were examined. The biochemical and molecular biological test results showed that BE had a good preventive effect on experimental acute liver injury. The experimental results provide a theoretical basis for further utilization of the blueberry resource.

## 2. Materials and Methods 

### 2.1. Extraction of BE

A total of 100 g of freeze-dried fresh blueberries were weighed and blended into a powder. The powder was added to 200 mL of a 45% ethanol solution, then the powder and ethanol solution were mixed, placed in water bath at 90 °C for 30 min for extraction and re-extracted one more time. The two extraction solutions were combined and then filtered. The blueberry polyphenol extract was obtained by rotating evaporation of the extraction solution after filtration [15].

### 2.2. Determination of the Polyphenol Content in BE

Different amounts of the chlorogenic acid standard were weighed and added to deionized water to prepare chlorogenic acid standard solutions with different concentrations. For each concentration, 1.0 mL of chlorogenic acid standard solution was pipetted into 25 mL flasks and then 3.0 mL of the Folin–Ciocalteu reagent was added and mixed well. After 5 min of reaction, 4.5 mL of a saturated Na_2_CO_3_ solution was added to the flask. After adding an equal volume of water, the reaction was carried out at 30 °C for 30 min in the dark. Finally, the absorbance value was measured at 747 nm and a chlorogenic acid standard curve was graphed [16]. BE was serially diluted to 10^−4^ and the absorbance values of BE were determined by the above method. The BE content was calculated according to the standard curve.

### 2.3. Mouse Experiment

Fifty 6-week-old specific pathogen-free (SPF) grade Kunming mice (male, body weight 20 ± 2 g) were used in the experiment. The experimental conditions were as follows: room temperature 25 °C, relative humidity of 60%, feed (basic feed) and drinking water ad libitum, cage padding changed every 2 day and adaptive feeding for 1 week. The mice were divided into five groups as follows with 10 mice per group: Normal group, model group, low concentration BE group (BEL group), high concentration BE group (BEH group) and silymarin group. The normal and model groups were intragastrically administered saline solution, the BEL and BEH mice were intragastrically administered 100 mg/kg and 200 mg/kg BE, respectively, and the silymarin group was intragastrically administered 200 mg/kg of silymarin; all treatments were performed for 14 days. On day 14, all mice except for those in the normal group were injected with the CCl_4_ inducer (CCl_4_ and olive oil at a volume ratio of 1:1, 0.1 mL/10 g) 1 hour after gavage administration [17]. After intraperitoneal injection of the CCl_4_ solution, all experimental mice were fasted for 24 h, and the liver and blood were collected. The liver tissue index was determined simultaneously as follows: liver tissue index = liver mass (g)/mouse body mass (kg) × 100. This study was conducted in accordance with the Declaration of Helsinki and the protocol was approved by the Ethics Committee of Chongqing Collaborative Innovation Center for Functional Food (201803001B).

### 2.4. Determination of the Serum Aspartate Aminotransferase (AST), Alanine Aminotransferase (ALT), Triglyceride (TG) and Total Cholesterol (TC) Levels

The collected blood samples were centrifuged at 4000 rpm for 10 min and the upper serum layer was collected. The serum AST, ALT, TG and TC levels were determined according to the kit instructions (Nanjing Jiancheng Bioengineering Institute, Nanjing, Jiangsu, China) [18].

### 2.5. Determination of The Serum Cytokine Interleukin-6 (IL-6), Tumor Necrosis Factor-α (TNF-α) and Interferon-γ (IFN-γ) Levels

The collected blood samples were centrifuged at 4000 rpm for 10 min and the upper serum layer was collected. The serum cytokine IL-6, TNF-α and IFN-γ levels were determined according to the kit instructions (Abcam, Cambridge, MA, USA) [18].

### 2.6. Determination of the SOD and TBARS Levels in Liver Tissue

The mouse liver samples were prepared as a 10% homogenate and centrifuged at 4000 rpm for 10 min. The supernatants were collected to determine the SOD and thiobarbituric acid reactive substances (TBARS) levels in the liver tissue according to the kit instructions (Nanjing Jiancheng Bioengineering Institute, Nanjing, Jiangsu, China) [18].

### 2.7. Pathological Observation of Liver Tissue

Liver tissue (0.5 cm^2^) was fixed in 10% formalin solution for 48 h. The liver tissue was dehydrated, cleared, immersed in wax, embedded, sliced and stained with haematoxylin and eosin (HE). The tissue morphological changes were observed under a light microscope (BX43, Olympus, Tokyo, Japan).

### 2.8. Quantitative PCR (qPCR) Assay

The tongue tissue of the mice was pulverized and total RNA in the tongue tissue was extracted using RNAzol. The extracted total RNA was diluted to 1 μg/μL and 5 μL of the diluted total RNA solution was used for the reverse transcription reaction according to the kit instructions to obtain the complementary DNA (cDNA) template. A total of 2 μL of the cDNA template was mixed with 10 μL of SYBR Green PCR Master Mix and 1 μL of the upstream and downstream primers (Thermo Fisher Scientific, Waltham, MA, USA) (Table 1). The qPCR reaction setting (StepOnePlus Real-Time PCR System, Thermo Fisher Scientific, Waltham, MA, USA) was as follows: 95 °C for 60 s, 40 cycles of 95 °C for 15 s, 55 °C for 30 s and 72 °C for 35 s and a final step of 95 °C for 30 s and 55 °C for 35 s. Glyceraldehyde-3-phosphate dehydrogenase (GAPDH) was used as an internal control. The 2^−ΔΔCt^ method was employed to calculate the relative gene expression levels [19].

### 2.9. Statistical Analysis

Three parallel experiments were performed for the serum and tissue assays for each mouse, and the average values were calculated. The data were analysed using the SAS 9.1 statistical software (SAS Institute Inc., Cary, NC, USA). One-way analysis of variance (ANOVA) was used to determine significant differences among the groups at the *p* < 0.05 level [20].

## 3. Results

### 3.1. Polyphenol Content in the BE Extract

**Figure 1:**
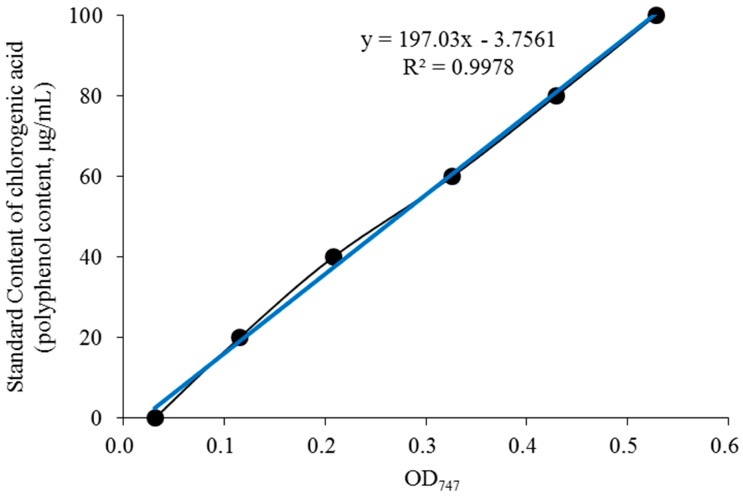
Standard curve of polyphenol content (chlorogenic acid).

The regression equation of the chlorogenic acid standard solution standard curve obtained in the experiment is Y = 197.03X − 3.7561 (Figure 1), where Y is the chlorogenic acid concentration and X is the absorbance value. The polyphenol content (chlorogenic acid) in BE was 52.7% according to the standard curve, indicating that the most main functioning component in the subsequent animal experiments was polyphenols.

### 3.2. The Body Weight, Liver Weight and Liver Index

As shown in Table 2, the body weights of the model group mice were lower than those of the other groups (*p* < 0.05), whereas the liver weights of the normal group mice were lower than those of the other groups. The liver index was highest for the model group mice and lowest for the normal group mice. The liver indexes of the mice with liver injury treated with BEH and silymarin were lower than that of the model group (*p* < 0.05).

### 3.3. Mouse Serum AST, ALT, TG and TC Levels

As shown in Table 3, the serum AST, ALT, TG and TC levels were lowest in the normal group mice and highest in the model group mice. After BE treatment, the AST, ALT, TG and TC levels were decreased in the mice with liver injury, and the effect was promoted by the increasing BE concentration. The effect of BE at 200 mg/kg (BEH) was not significantly different from that of silymarin (*p* > 0.05).

### 3.4. Serum Cytokine IL-6, TNF-α and IFN-γ Levels

As shown in Table 4, the serum cytokine IL-6, TNF-α and IFN-γ levels were lowest in the normal group mice and significantly increased after CCl_4_-induced liver injury. The BE and silymarin treatments significantly inhibited the increase in the cytokine levels caused by liver injury (*p* < 0.05). The IL-6, TNF-α and IFN-γ levels were lower in the silymarin treatment group mice than in the BEH treatment group mice, whereas the cytokine levels in the BEL treatment group mice were only lower than those in the model group.

### 3.5. SOD Activity and TBARS Level in Mouse Liver Tissue

Table 5 shows that the SOD activity was lowest and the TBARS level was highest in the liver tissue of the model group mice, whereas the liver tissue of the normal group showed the opposite trend, with the highest SOD activity and the lowest TBARS level. BE treatment restored the SOD activity and TBARS level in the liver tissue of the mice with liver injury to values close to those of the normal group, with the higher concentration having a more obvious effect. The high BE concentration had an effect similar to that of the drug silymarin.

### 3.6. Pathological Observation of Mouse Liver

**Figure 2:**
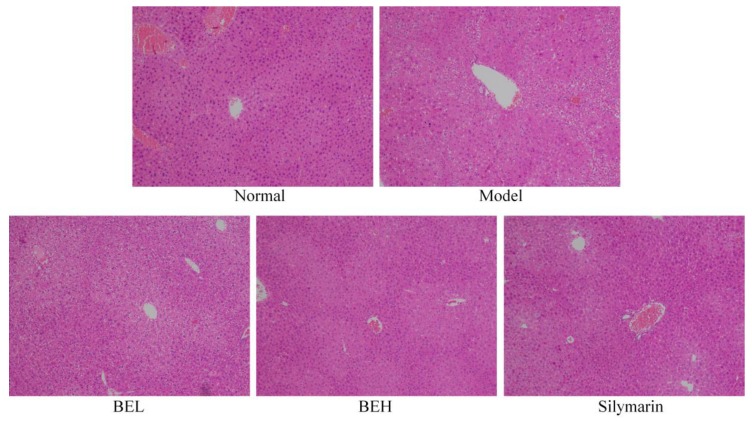
H&E pathological observation of liver in mice. Magnification 100×. BEL: mice treated with low concentration of blueberry polyphenols (100 mg/kg); BEH: mice treated with high concentration of blueberry polyphenols (200 mg/kg); Silymarin: mice treated with 200 mg/kg silymarin.

Figure 2 shows that the liver lobule structure of the normal group mice was clear, the hepatocytes were arranged in a radial manner centred on the central vein, the hepatocytes were free of degeneration and necrosis and no inflammatory cell infiltration was visible in the portal area. The hepatocytes of the model group mice showed diffuse oedema and fat degeneration and the hepatocytes around the central vein demonstrated massive necrosis. Compared with that of the BEL group, the necrotic area in the BEH group was smaller and some hepatocytes were still swelling. The liver tissue structure of the BEH and silymarin groups was normal, but hepatocyte oedema was still visible. Most of the liver cells showed no obvious necrosis and only a small portion of the liver tissue showed dotted hepatocyte necrosis; however, the degree of oedema was significantly reduced compared with that of the model group. No balloon-like changes were detected and the degree of hepatocyte necrosis was significantly reduced.

### 3.7. Cu/Zn-SOD mRNA Expression in Mouse Liver Tissue

**Figure 3:**
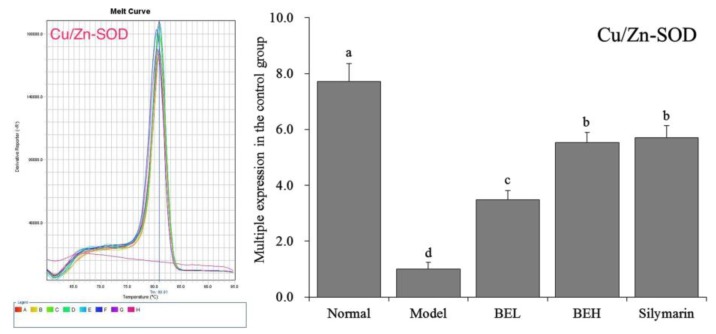
The Cu/Zn-SOD mRNA expression in liver of mice. ^a–^^d^ Mean values with different letters in the same bar are significantly different (*p* < 0.05) according to Duncan’s multiple-range test. BEL: mice treated with low concentration of blueberry polyphenols (100 mg/kg); BEH: mice treated with high concentration of blueberry polyphenols (200 mg/kg); Silymarin: mice treated with 200 mg/kg silymarin.

Figure 3 showed that Cu/Zn-SOD expression was lowest in the model group mice. After BE treatment, Cu/Zn-SOD expression was significantly increased in the liver tissue of the mice with liver injury (*p* < 0.05). The effect of BEH was better than that of BEL and was similar to that of silymarin.

### 3.8. Mn-SOD mRNA Expression in Mouse Liver Tissue

**Figure 4:**
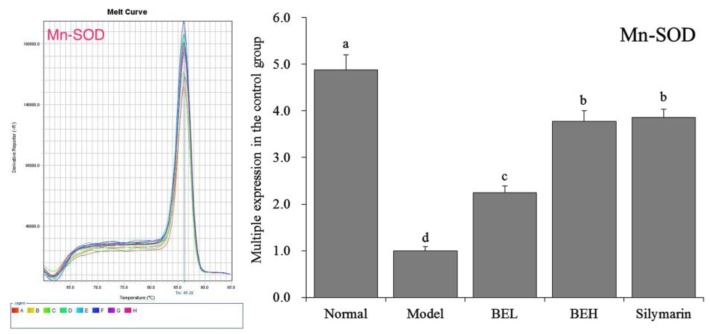
The Mn-SOD mRNA expression in liver of mice. ^a–d^ Mean values with different letters in the same bar are significantly different (*p* < 0.05) according to Duncan’s multiple-range test. BEL: mice treated with low concentration of blueberry polyphenols (100 mg/kg); BEH: mice treated with high concentration of blueberry polyphenols (200 mg/kg); Silymarin: mice treated with 200 mg/kg silymarin.

Figure 4 shows that Mn-SOD mRNA expression in the liver tissue was highest in the normal group mice and decreased significantly after CCl_4_-induced liver injury (*p* < 0.05). Both BE and silymarin significantly (*p* < 0.05) inhibited the decrease in Mn-SOD expression in the liver tissue induced by CCl_4_ and the effect of BE in enhancing Mn-SOD expression became stronger with increasing concentration.

### 3.9. CAT mRNA Expression in Mouse Liver Tissue

**Figure 5:**
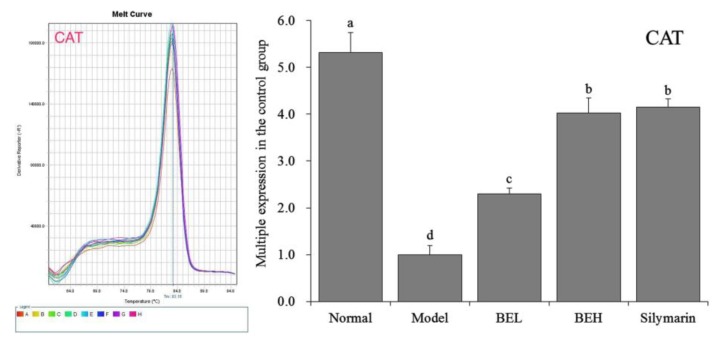
The CAT mRNA expression in liver of mice. ^a–d^ Mean values with different letters in the same bar are significantly different (*p* < 0.05) according to Duncan’s multiple-range test. BEL: mice treated with low concentration of blueberry polyphenols (100 mg/kg); BEH: mice treated with high concentration of blueberry polyphenols (200 mg/kg); Silymarin: mice treated with 200 mg/kg silymarin.

Figure 5 showed that CAT mRNA expression in the liver tissue was significantly higher in the normal group than in the other groups (*p* < 0.05). The CAT expression levels in the liver tissues of the BEH and silymarin group mice were lower than that in the normal group, and no significant difference in CAT expression was found between the two groups (*p* > 0.05), although the levels were higher than those of the BEL and model group mice.

### 3.10. COX-2 mRNA Expression in Mouse Liver Tissue

**Figure 6:**
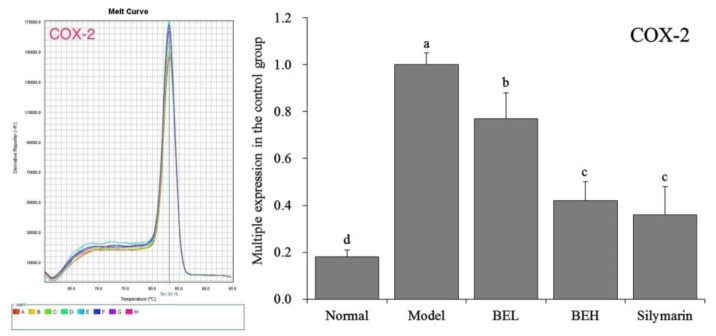
The COX-2 mRNA expression in liver of mice. ^a–d^ Mean values with different letters in the same bar are significantly different (*p* < 0.05) according to Duncan’s multiple-range test. BEL: mice treated with low concentration of blueberry polyphenols (100 mg/kg); BEH: mice treated with high concentration of blueberry polyphenols (200 mg/kg); Silymarin: mice treated with 200 mg/kg silymarin.

Figure 6 shows that COX-2 mRNA expression in the liver tissue of the normal group of mice demonstrated an opposite trend compared to the Cu/Zn-SOD, Mn-SOD and CAT expression levels and was significantly lower than those of the other groups (*p* < 0.05). COX-2 expression in the liver tissue was highest in the model group mice. BE significantly affected the abnormal COX-2 expression in the liver tissue caused by CCl_4_ (*p* < 0.05) and decreased COX-2 expression in liver tissue to a level close to that of the normal group mice. At the same concentration, the effects of BE and silymarin were not significantly different (*p* > 0.05).

## 4. Discussion

The liver is one of the most important organs of the human body. Liver damage affects the health of the body and even endangers life. At present, the liver and serum function indexes are used clinically to evaluate liver lesions. The liver index, which is also known as the liver coefficient, is one pathological indicator that is used to measure liver damage and has been widely applied to evaluate the extent of experimental liver injury; changes in the liver index can directly reflect the extent of liver damage in experimental animals [21]. Therefore, determination of the liver index of mice can directly reflect the structural changes and functions of the liver and can be applied to evaluate the degree of liver damage in mice with liver injury. The results of this study also showed that CCl_4_ increased the organ index in mice and that BE effectively alleviated the increase and even decreased the liver index of mice with liver injury to a level close to that of normal mice. The effect of the BE was similar to that of silymarin, which is a drug for liver injury.

AST and ALT are the most sensitive indicators for the diagnosis of liver cell damage. During amino acid synthesis and catabolism, AST and ALT play vital roles as endo-enzymes in hepatocytes. Under the circumstance of the normal working condition of the body, ALT and AST levels in the blood are very low and, thus, the activity of these two enzymes in normal serum is very low. When the liver tissue is damaged and the cell membrane permeability increases, these two enzymes penetrate into the blood in large quantities, leading to a significant increase in the activity of the enzymes in the sera. Therefore, an increase in serum AST and ALT can reflect the extent of liver cell damage [22]. When CCl_4_ enters the animal body, liver microsomal lipids and hepatocyte membrane phospholipid molecules are attacked by free radicals generated by CCl_4_, which in turn trigger changes in the TC and TG levels in the liver [23]. The increase in the AST, ALT and TBIL levels indicates an exaggeration of liver damage. The experimental data from this study also confirmed that CCl_4_ resulted in an increase in the AST, ALT, TC and TG levels in mice, whereas BE led to a significant reduction of the same levels in the serum and thus exerted a preventive effect on liver damage.

TNF-α is a polypeptide mediator with a wide range of biological activities that mediates liver damage resulting from various causes. Liver damage is directly related to the increase in TNF-α [24]. IL-6 can stimulate the synthesis of acute phase proteins in hepatocytes to participate in the inflammatory response. IL-6 can also effectively promote the cachexia induced by TNF and IL-1 and, thus, exaggerate the degree of tissue damage [25]. IFN-γ can mediate the damage to non-target cells caused by intracellular viruses and participate in the injury response with TNF-α to promote liver damage and the liver tissue inflammatory response [26]. This study confirmed that BE could prevent and reduce liver damage by reducing the IL-6, TNF-α and IFN-γ levels in mice.

CCl_4_ causes oxidative stress in mouse liver tissue, resulting in the production of many free radicals [27]. SOD activity in the liver tissue can be used to evaluate the extent of liver damage. Regulated enhancement of SOD activity is the main mechanism of enzymatic antioxidation in the body; therefore, SOD activity reflects the liver tissue damage to a certain extent [28]. TBARS is the metabolic end product of oxidative damage and accumulates in the body after liver injury. The TBARS level can reflect the degree of cell damage caused by free radical attack. Therefore, TBARS is also a sensitive indicator of liver injury [29]. In this study, BE enhanced SOD activity and reduced the TBARS level in the liver tissue of mice with liver injury, thereby protecting the liver from the damage caused by oxidative stress induced by CCl_4_.

SOD is classified according to its different metal prosthetic groups. Cu/Zn-SOD containing the Cu and Zn metal prosthetic groups is the most common enzyme and mainly resides in the cytoplasm. Mn-SOD containing the Mn metal prosthetic group is localized in the mitochondria of eukaryotic cells and in prokaryotic cells [30]. As the major SOD isoenzyme in the human body, Cu/Zn-SOD is widely distributed in the extracellular matrix and on the cell surface. Its main function is to remove extracellular O^2−^. Mn-SOD is also a SOD isoenzyme that functions to eliminate O^2−^ [31]. CAT is an enzyme scavenger that decomposes hydrogen peroxide into molecular oxygen and H_2_O and thereby removes hydrogen peroxide from the body to protect cells from toxicity. It can also alleviate the tissue inflammation and damage caused by oxidative stress [32]. COX-2 promotes inflammatory reactions and causes tissue damage and is one of the key enzymes that cause inflammatory reactions [33]. COX-2 can induce the production of many inflammatory cytokines, including TNF-α, which in turn can interact with COX-2 to aggravate liver damage. Moreover, COX-2 can play a positive role under the state of oxidative stress in the liver and ROS can cause tissue damage by regulating COX-2 to induce inflammatory reactions [34]. In this study, determination of the SOD, CAT and COX-2 mRNA levels in mouse liver revealed that BE could effectively inhibit COX-2 expression resulting from oxidative stress in the liver tissue of mice with liver injury. Thus, BE protected the liver by inhibiting oxidative stress through increasing the Cu/Zn-SOD, Mn-SOD and CAT expression levels.

In this study, we preliminarily found that blueberry polyphenols have good biological activity and extracting more polyphenols from blueberry is also the focus of making better use of this resource. Hydrodynamic cavitation has been applied as a technology in the extraction of active substances, which can effectively improve the extraction rate of active substances [35]. The application of this technology in the extraction of blueberry polyphenols can also become a research focus in the future.

## 5. Conclusions

This study shows that BE can inhibit inflammation and oxidative stress in mice with liver injury, regulate liver function indexes and inflammatory cytokine levels, modulate the mRNA expression levels of genes related to oxidative stress and inflammation in liver tissue and, thereby, comprehensively prevent the liver injury caused by CCl_4_. Therefore, BE is rich in polyphenols, it has good liver protection function and has the value of further development and utilization. In this study, the role of BE is verified using basic animal experiments and the biological activity of BE awaits comprehensive validation by detailed human experiments in the future.

## Figures and Tables

**Table 1:** Sequences of primers used in this study.

Gene Name	Sequence
Cu/Zn-SOD	Forward: 5′-AACCAGTTGTGTTGTCAGGAC-3′
	Reverse: 5′-CCACCATGTTTCTTAGAGTGAGG-3′
Mn-SOD	Forward: 5′-CAGACCTGCCTTACGACTATGG-3′
	Reverse: 5′-CTCGGTGGCGTTGAGATTGTT-3′
CAT	Forward: 5′-GGAGGCGGGAACCCAATAG-3′
	Reverse: 5′-GTGTGCCATCTCGTCAGTGAA-3′
COX-2	Forward: 5′-GGTGCCTGGTCTGATGATG–3′
	Reverse: 5′-TGCTGGTTTGGAATAGTTGCT–3′
GAPDH	Forward: 5′-AGGTCGGTGTGAACGGATTTG-3′
	Reverse: 5′-GGGGTCGTTGATGGCAACA-3′

Cu/Zn-SOD: cuprozinc-superoxide dismutase; Mn-SOD: manganese superoxide dismutase; CAT: catalase; COX-2: cyclooxygenase 2; GAPDH: glyceraldehyde-3-phosphate dehydrogenase.

**Table 2:** Effects of blueberry polyphenols on body weight, liver weight and liver index of mice with hepatic injury induced by CCl_4_ (*N* = 10).

Group	Body Weight (g)	Liver Weight (g)	Liver Index
Normal	46.58 ± 1.42 ^a^	1.94 ± 0.22 ^b^	4.16 ± 0.3 ^c^
Model	37.11 ± 1.31 ^b^	2.20 ± 0.22 ^a^^b^	5.93 ± 0.4 ^a^
BEL	45.87 ± 1.12 ^a^	2.44 ± 0.38 ^a^	5.32 ± 0.4 ^a^^b^
BEH	46.63 ± 1.08 ^a^	2.25 ± 0.24 ^a^^b^	4.83 ± 0.3 ^b^
Silymarin	47.26 ± 1.22 ^a^	2.18 ± 0.17 ^a^^b^	4.61 ± 0.4 ^b^

Values presented are the mean ± standard deviation (*N* = 10/group). ^a–^^c^ Mean values with different letters over the same column are significantly different (*p* < 0.05) according to Duncan’s multiple range test. BEL: mice treated with low concentration of blueberry polyphenols (100 mg/kg); BEH: mice treated with high concentration of blueberry polyphenols (200 mg/kg); Silymarin: mice treated with 200 mg/kg silymarin.

**Table 3:** The levels of AST, ALT, TG and TC in serum of mice (*N* = 10).

Group	AST (U/L)	ALT (U/L)	TC (mg/dL)	TG (mg/dL)
Normal	12.37 ± 3.85 ^d^	5.36 ± 0.87 ^d^	108.32 ± 5.22 ^d^	5265.32 ± 87.35 ^d^
Model	149.83 ± 18.32 ^a^	28.91 ± 3.36 ^a^	635.28 ± 31.88 ^a^	16387.20 ± 233.15 ^a^
BEL	109.86 ± 5.25 ^b^	19.32 ± 3.08 ^b^	487.36 ± 25.87 ^b^	11538.69 ± 256.32 ^b^
BEH	58.36 ± 4.82 ^c^	11.18 ± 2.12 ^c^	245.23 ± 19.68 ^c^	8325.05 ± 315.24 ^c^
Silymarin	53.20 ± 6.22 ^c^	10.65 ± 2.31 ^c^	239.58 ± 23.89 ^c^	8158.95 ± 298.23 ^c^

Values presented are the mean ± standard deviation (*N* = 10/group). ^a–^^d^ Mean values with different letters over the same column are significantly different (*p* < 0.05) according to Duncan’s multiple range test. BEL: mice treated with low concentration of blueberry polyphenols (100 mg/kg); BEH: mice treated with high concentration of blueberry polyphenols (200 mg/kg); Silymarin: mice treated with 200 mg/kg silymarin. AST: Aspartate Aminotransferase; ALT: Alanine Aminotransferase TG: Triglyceride; TC: Total Cholesterol.

**Table 4:** The cytokine levels of IL-6, TNF-α and IFN-γ in serum of mice (*N* = 10).

Group	IL-6 (pg/mL)	TNF-α (pg/mL)	IFN-γ (pg/mL)
Normal	23.01 ± 2.13 ^d^	312.65 ± 11.08 ^e^	39.12 ± 1.52 ^d^
Model	59.33 ± 3.87 ^a^	733.48 ± 29.38 ^a^	81.30 ± 4.72 ^a^
BEL	46.30 ± 2.81 ^b^	541.69 ± 25.63 ^b^	65.17 ± 5.25 ^b^
BEH	33.05 ± 2.26 ^c^	409.71 ± 16.78 ^c^	51.36 ± 3.97 ^bc^
Silymarin	30.87 ± 3.01 ^c^	367.97 ± 11.36 ^d^	47.36 ± 3.32 ^c^

Values presented are the mean ± standard deviation (*N* = 10/group). ^a–^^e^ Mean values with different letters over the same column are significantly different (*p* < 0.05) according to Duncan’s multiple range test. BEL: mice treated with low concentration of blueberry polyphenols (100 mg/kg); BEH: mice treated with high concentration of blueberry polyphenols (200 mg/kg); Silymarin: mice treated with 200 mg/kg silymarin. IL-6: interleukin-6; TNF-α: tumor necrosis factor-α; IFN-γ: interferon-γ.

**Table 5:** The levels of SOD and TBARS in hepatic tissue of mice (*N* = 10).

Group	SOD (U/mg)	TBARS (nmol/mg)
Normal	126.86 ± 8.33 ^a^	1.51 ± 0.44 ^d^
Model	28.39 ± 6.28 ^d^	4.83 ± 0.52 ^a^
BEL	69.35 ± 5.59 ^c^	3.75 ± 0.31 ^b^
BEH	92.79 ± 5.36 ^b^	2.27 ± 0.25 ^c^
Silymarin	95.02 ± 6.30 ^b^	2.11 ± 0.29 ^c^

Values presented are the mean ± standard deviation (*N* = 10/group). ^a–^^d^ Mean values with different letters over the same column are significantly different (*p* < 0.05) according to Duncan’s multiple range test. BEL: mice treated with low concentration of blueberry polyphenols (100 mg/kg); BEH: mice treated with high concentration of blueberry polyphenols (200 mg/kg); Silymarin: mice treated with 200 mg/kg silymarin; SOD: superoxide dismutase; TBARS: thiobarbituric acid reactive substances
